# Metaverse in Healthcare Integrated with Explainable AI and Blockchain: Enabling Immersiveness, Ensuring Trust, and Providing Patient Data Security

**DOI:** 10.3390/s23020565

**Published:** 2023-01-04

**Authors:** Sikandar Ali, Abdullah, Tagne Poupi Theodore Armand, Ali Athar, Ali Hussain, Maisam Ali, Muhammad Yaseen, Moon-Il Joo, Hee-Cheol Kim

**Affiliations:** 1 Department of Digital Anti-Aging Healthcare, Inje University, Gimhae 50834, Republic of Korea; 2 Institute of Digital Anti-Aging Healthcare, College of AI Convergence, u-AHRC, Inje University, Gimhae 50834, Republic of Korea

**Keywords:** metaverse, healthcare, explainable AI, blockchain, healthcare metaverse, immersive technologies, mixed reality, digital twins, immersive technologies in healthcare, NLP

## Abstract

Digitization and automation have always had an immense impact on healthcare. It embraces every new and advanced technology. Recently the world has witnessed the prominence of the metaverse which is an emerging technology in digital space. The metaverse has huge potential to provide a plethora of health services seamlessly to patients and medical professionals with an immersive experience. This paper proposes the amalgamation of artificial intelligence and blockchain in the metaverse to provide better, faster, and more secure healthcare facilities in digital space with a realistic experience. Our proposed architecture can be summarized as follows. It consists of three environments, namely the doctor’s environment, the patient’s environment, and the metaverse environment. The doctors and patients interact in a metaverse environment assisted by blockchain technology which ensures the safety, security, and privacy of data. The metaverse environment is the main part of our proposed architecture. The doctors, patients, and nurses enter this environment by registering on the blockchain and they are represented by avatars in the metaverse environment. All the consultation activities between the doctor and the patient will be recorded and the data, i.e., images, speech, text, videos, clinical data, etc., will be gathered, transferred, and stored on the blockchain. These data are used for disease prediction and diagnosis by explainable artificial intelligence (XAI) models. The GradCAM and LIME approaches of XAI provide logical reasoning for the prediction of diseases and ensure trust, explainability, interpretability, and transparency regarding the diagnosis and prediction of diseases. Blockchain technology provides data security for patients while enabling transparency, traceability, and immutability regarding their data. These features of blockchain ensure trust among the patients regarding their data. Consequently, this proposed architecture ensures transparency and trust regarding both the diagnosis of diseases and the data security of the patient. We also explored the building block technologies of the metaverse. Furthermore, we also investigated the advantages and challenges of a metaverse in healthcare.

## 1. Introduction

Recently the metaverse has gained much popularity and attention all around the world. Now the world is shifting towards the metaverse which enables mankind to link the physical world with the virtual world in a more immersive way. The metaverse can perform a variety of activities with the help of avatars that act similarly to real human beings in the virtual world. Metaverse technology empowers human beings to build a digital world while transforming a better physical world. Although the term metaverse is not a new term, nowadays it has become a buzzword. Neal Stephenson, a writer who wrote “Snow Crash” coined the word metaverse in his science fiction novel for the first time in 1992 [1]. The word “metaverse” is the combination of two words, “meta” means transcending and “verse” pertains to the universe describing the virtual world linked to the physical world. It is a convergence of technologies that facilitates the physical world to interact with the virtual environments digitally via augmented reality (AR), virtual reality (VR), extended reality, and mixed reality (MX). Furthermore, the metaverse is considered as a web of a virtual world where avatars interact in the virtual world enabling an immersive experience. Metaverse incorporates different kinds of new technologies for example artificial intelligence (AI), blockchain, robotics, computer vision, the Internet of Things (IoT), cloud/Edge computing, etc. Fusing digital twin technology it forms a mirror image of the real world, an immersive experience is achieved with augmented reality, a strong economic system can be built based on blockchain technology, and highly accurate and precise predictive systems are developed based on artificial intelligence technology. Metaverse is in its nascent stage and is continuously evolving. It is going to open new avenues for all sectors be it healthcare [2,3], education [4], tourism industry [5], agriculture [6], social networking, entertainment, or gaming [7,8]. Considering the potential of the metaverse, the giant social media company, Facebook changed its name to Meta which reinforced the importance of the metaverse [9]. The CEO of the company is determined to bring innovation to business with the help of the metaverse. The metaverse has also revolutionized the gaming world. Many companies, for example, Fortnite by Epic games [10], have been using metaverse enabling the players to go into a virtual world and enjoy playing the game virtually.

### Motivation

Like other domains, the medical domain also carries an encouraging future in the metaverse [3,11]. Metaverse has enough potential to leverage the healthcare sector by facilitating healthcare services virtually with an immersive experience powered by its amazing features [12]. In the wake of the COVID-19 pandemic, the need for virtual and non-face-to-face treatment received much popularity. Moreover, it has changed the course of life in one way or another. Nowadays patients are being treated virtually through nurse avatars [13]. Patients’ data, symptoms, and other necessary information are collected using wearable sensors. Patients are questioned by the avatars, such as Sensely and NHS to gather complete information about the patients and their medical history [14]. The data are processed and analyzed by the models powered by artificial intelligence and blockchain. The patients are treated by the physicians by getting feedback from the AI-powered models. Several technological domains are involved to accomplish the metaverse healthcare ecosystem. For example, fifth generation (5G) internet, artificial intelligence, digital twins, blockchain, etc., are the building blocks of metaverse ecosystems.

On one hand, the metaverse provides many facilities and benefits; however, on the other hand, there are several challenges as well for example network security, data security, privacy issues, etc. Metaverse systems have greater potential for sensitive data collection than conventional systems, which poses a serious threat to user privacy. There is a high chance of private data in the virtual environment, metaverse platform, or service system being altered or leaked. For instance, while a user is utilizing the platform, an avatar’s data, such as voice and video recordings, could be eavesdropped, or an attacker could create a fake avatar and use it maliciously. Therefore, the security and privacy of users’ data should not be compromised. We try to provide solutions to this issue by integrating blockchain technology into the metaverse system to secure healthcare data.

There are several potential benefits of using metaverse in healthcare. First, the patients can get medical facilities at their homes and can examine patients virtually but with an immersive experience as if they are face-to-face with each other in the same environment. Patients with disability and home-bound patients would receive more benefits. Second, metaverse offers quick delivery of healthcare resources and services. This will, consequently, reduces the mortality rate of patients due to delay in getting emergency healthcare services. Third, patients from remote areas where there is a shortage of healthcare professionals and doctors can receive benefits regardless of distance and travel challenges. Fourth, it reduces clinic visits from patients slashing the carbon footprint due to transport and helps prevent contagious diseases and pandemics such as COVID-19. Furthermore, it decreases healthcare service costs. Fifth, metaverse also offers seamless patient monitoring functionalities through AR and VR technologies without geographical barriers. Sixth, it is used for reducing patients’ stress and anxiety. Doctors and physicians use it for modeling surgeries and training for challenging procedures before they attempt them on real patients. Moreover, different kinds of chronic diseases, phobias, and mental health problems are being treated with metaverse technology. The goal of this research work is to build a framework that will help to carry out virtual treatments in the metaverse using the latest technologies such as artificial intelligence, and blockchain in combination with immersive technologies.

The contributions of our research work are as follows:

- This paper presents the potential of metaverse fusing with artificial intelligence and blockchain in the healthcare sector.
- This study highlights the building block technologies that make up the metaverse while demonstrating their role in metaverse healthcare.
- This work proposes a metaverse-based healthcare system by integrating explainable artificial intelligence and blockchain for the diagnosis and treatment of diseases.
- To ensure explainability, Grad-Cam and Lime techniques are used while data security is ensured by using decentralized blockchain technology.
- Furthermore, this study also identifies the advantages and challenges of the metaverse in the healthcare sector.

This research work is organized as follows: Section 1 contains the introduction of the metaverse, the motivation for this study, and the potential benefits of using the metaverse in healthcare. Section 2 describes the related work on metaverse, artificial intelligence, and blockchain. Section 3 deals with the building block technologies and framework of the metaverse. In Section 4, our proposed metaverse-based healthcare system architecture is presented in detail. Section 5 presents the advantages and challenges of the metaverse. In Section 6, we conclude our research work.

## 2. Related Works

Metaverse technology is gaining popularity day by day and it is considered to be the next level of digital evolution. It has the potential to bring improvement in every domain such as healthcare, entertainment, business, education, etc. It is an amazing technology that combines other technologies as well and offers a virtual world with an immersive experience. Nowadays researchers are focusing on this new emerging technology and there is a plethora of related research works on metaverse technology. The researchers have investigated the role of the metaverse in different fields such as healthcare [15], medicine [16], e-commerce [17], education [18], and smart cities [19,20].

Ioannis Skalidis et al. [21] presented an idea of a metaverse-based cardiovascular medicine. They termed it as CardioVerse. It can transform and reshape the treatment procedure for cardiovascular disease. It has several other applications such as improving the efficiency and precision of cardiovascular interventions, diagnosis, and prevention of disease, etc. Chayakrit Krittanawong MD et al. [22] discussed the integration of artificial intelligence and blockchain in cardiovascular medicine. They elaborated on the potential future directions and applications of these technologies in the field of cardiovascular disease. Furthermore, they evaluated the challenges and explored novel solutions for better treatment and prevention of cardiovascular disease. Metaverse has been used for training and education purposes as well. Huilyung Koo et al. [23] conducted thoracic surgery in a smart operating room of Seoul National University (SNU) Bundang Hospital. This surgery was broadcast and attended by more than 200 surgeons. The thoracic surgeons received online training while wearing a head-mounted display (HMD) with an immersive experience. The participants could participate in the discussion and view the surgical procedures in the virtual environment. Moreover, Cancer, which is one of the most deleterious diseases in the world, is being treated using metaverse technology. Using augmented reality, Johns Hopkins was able to remove the patient’s cancerous tumor from the spine of the patient successfully [24]. Jane Thomason [2] investigated the use of metaverse for the prevention and management of obesity and chronic diseases. In his research work, he has discovered the possibilities of metaverse for chronic disease. Through avatars and wearable technologies, patients can receive consultancy, initial diagnosis, personalized care, social prescribing, and patient education. Gamification functionality in the metaverse can mobilize people regarding their well-being and balanced health condition. Desiderio Cano Porras et al. [25] systematically reviewed the VR-based treatment for patients with Parkinson’s disease. They included ninety-seven research articles in their study and conducted detailed and comprehensive research. They concluded that the VR-based rehabilitation method is one of the best treatment methods for Parkinson’s disease. Zhen Liu et al. [26] highlighted the importance of VR-aided therapy for different kinds of medical conditions. VR-based technologies overcome the limitations of face-to-face traditional therapies by providing facilities remotely. During COVID-19, VR-based therapies emerged as the most convenient way of treatment for patients. Ali Garavand et al. [15] presented the phenomenon of the metaverse and their impact on health. They collected data through electronic searches from famous scientific databases for example PubMed, Web of Science, etc. They concluded that the metaverse is expanding exponentially in various fields of healthcare such as medicine, medical interventions, medical imaging, etc.

Bokyung kye et al. [27] investigated the possibilities and limitations of educational applications of the metaverse. They divided the metaverse roadmap into four categories, e.g., virtual reality, augmented reality, mirror world, and lifelogging. They stated that an augmented reality T-shirt helps medical students to understand the human anatomy in a better way compared to the conventional ways of education. Equally, there are a few limitations of metaverse applications such as weaker social connections, privacy issues, etc. Neama A. Dahan et al. [28] proposed a metaverse framework for an e-learning environment. This framework consists of infrastructure, avatars, cloud-based applications, and other necessary technologies, e.g., blockchain, virtual reality, and augmented reality. Md Ariful Islam Mozumder et al. [29] explored the technological roadmap of the metaverse. They discussed the fundamental technologies and techniques of a metaverse in detail. Furthermore, they investigated the medical domain where metaverse could be helpful and improve overall healthcare services. Athar A. et al. [30] investigated the role of artificial intelligence and blockchain in the metaverse. In their research work, they illustrated the position of AI, blockchain and other metaverse building block technologies using a metaverse layered architecture. Moreover, they highlighted some key use cases of AI and blockchain in the metaverse in domains such as healthcare, smart manufacturing, and education.

Syed Badruddoja et al. [31] developed blockchain by integrating it with artificial intelligence. They used smart contracts on the Ethereum blockchain. Their AI model achieved an accuracy of 95%. Furthermore, they delineated the challenges of metaverse development for smart contracts using artificial intelligence. Ayesha Shahnaz et al. [32] presented a blockchain framework for the electronic health record (EHR). They establish granular access controls to guarantee the secure storage of electronic records of their proposed framework. They explored the scalability and security of their proposed architecture. Table 1 summarizes the comparative analysis of some of the use cases of the healthcare metaverse.

## 3. Metaverse Framework and Building Block Technologies

**Figure 1:**
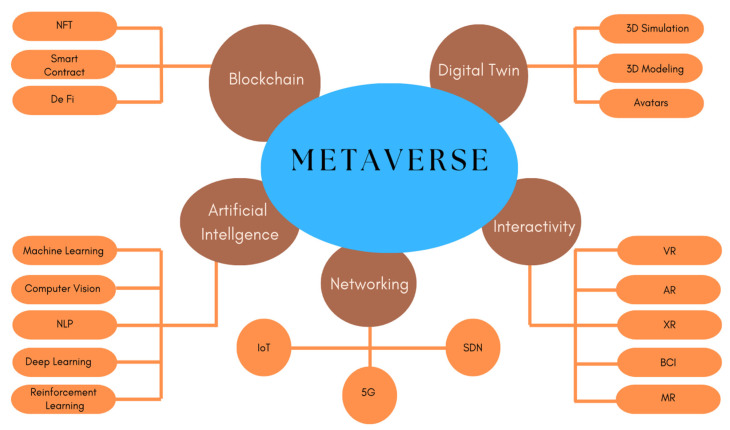
Building block technologies of the metaverse.

Metaverse is an interdisciplinary ecosystem created by embedding different other technologies at different layers of its whole architecture. It is a 3D version of the current internet. In a metaverse environment, several components act to interact between the physical and virtual worlds. Among them, users are one of the key components. A user can interact with the virtual worlds using some devices for example AR/VR glasses or head-mounted displays (HMDs). These devices enable users to interact and perform different actions virtually. IoT networks, virtual service providers (VSPs), and physical service providers (PSPs) are also some of the key components used for the interaction between the real world and the virtual world. The data are collected from the real world through the IoT and sensor networks and are used to develop digital twins. The virtual service providers (VSPs) and physical service providers (PSPs) help maintain the virtual and real-world environments of the metaverse. Some of those indispensable technologies are discussed below and shown in Figure 1.

### 3.1. Virtual Reality (VR)

Virtual reality (VR) offers a simulated experience to users using a head-mounted display (HMD) or VR glasses [39]. It allows users to immerse themselves in a 3D digital world using software and hardware components. Moreover, it provides advanced technical capabilities and a seamless immersive experience allowing freedom of moment to the users in the virtual world [40]. Although VR technology is not a new concept, it gained much attention after the advent of the metaverse. The investment in VR startups was USD 2.3 billion in 2016, now it is expected to reach USD 20.9 billion by 2025 [41]. The tech giants, for example, Google, Meta (previously Facebook), Apple, HTC, Sony, etc., are dealing with VR devices such as haptic gloves, optical trackers, 3D mice, motion controllers’ omnidirectional treadmills (ODts), etc. These devices have amazing functionalities for example Oculus Quest 2 has built-in processors and is a kind of all-in-one VR platform. Many countries such as South Korea, China, the USA, and Japan are focusing on the VR industry as a key technology direction that has the potential to boost their overall economy [42]. In the healthcare sector, VR technology is being used for surgery, physical therapy, stress and pain reduction, cognitive rehabilitation, etc. [43].

### 3.2. Extended Reality (XR)

Extended reality is an umbrella term that refers to VR, AR, and mixed reality (MR). It includes all virtual and real combined environments. The term extended reality was used in 1960 for the first time in history [44]. Later on, this technology is used widely in almost every industry such as healthcare, education, manufacturing, mining, etc [45,46]. It offers myriad varieties of applications and a huge number of levels in a virtual environment. In the healthcare domain, three different kinds of VR technologies have been used namely: haptic device simulators, head-mounted displays (HMDs), and computer-based simulations [47]. Among these three technologies, haptic simulators are very popular and most often used in surgeries and other treatments for patients.

### 3.3. Augmented Reality (AR)

Augmented reality is also one of those technologies that have transformed the outlook of the world. The users’ real-world views are strengthened by augmented reality (AR) with digital overlays that blend artificial objects. In other words, augmented reality means overlaying digital images onto the real world to enhance them with digital details. One of the best examples of augmented reality is Pokemon Go which enables us to see the world around us through our smartphones and can superimpose digital characters. Likewise, we use several kinds of filters for our social media photos on Snapchat to augment our looks, for example, bunny ears, different types of makeup, etc. Doctors and surgeons use augmented reality for surgery that augments the part of the body where it needs the procedure and assists them throughout the procedure [48]. The visualization is enhanced by superimposing 3D images of patients on real views of the surgical field. It helps save the lives of patients with high accuracy. Google Glass, Microsoft’s HoloLens, and the Magic Leap are some of the famous AR devices.

### 3.4. Internet of Things (IoT) and Network

The Internet of Things (IoT) offers a wide range of technologies such as sensors, wireless networks, and nanotechnology, to connect and communicate between a large number of devices such as smartphones, smart watches, healthcare devices, etc [49]. IoT along with other technologies is transforming the lives of human beings while making things easier for us. Thus, enhances the quality of our life. It is widely used in healthcare providing facilities to patients and physicians. Patients can be monitored remotely via different kinds of IoT devices thus increasing healthcare quality and decreasing healthcare costs [50]. This technology is also an integral part of the metaverse ecosystem. The capabilities of the metaverse are enhanced by integrating IoT technology [51]. The IoT devices are incentivized to gather and sense the physical status of the objects which will help virtual service providers to synchronize the digital twins [52].

### 3.5. Edge/Cloud Computing

Edge/Cloud computing is a new concept in the computing domain where computing operations are performed at the edge of the network. This technology aims to provide computing services closer to the origin of data [53]. With the development of the Internet of Things (IoT) and edge devices, the growth of data volume is increasing every passing day. Thus, posing several challenges such as energy consumption, privacy and security of data, and delay in real-time operations. To overcome these challenges edge computing concept was introduced which is a new computing mode closer to the edge of the network devices. It enhances security and privacy, data optimization, and real-time business [54].

### 3.6. Artificial Intelligence (AI)

Artificial intelligence (AI) is a well-known technology that has been used in every field from manufacturing industries to agriculture, healthcare to human resources, and gaming to businesses [55,56,57,58,59]. Artificial intelligence enables machines to mimic the human brain and it learns from the experience just like a human does. Based on experience, it performs different kinds of tasks. AI includes machine learning, deep learning, natural language processing, computer vision, and many other subfields. Machine learning, which is one of the wide areas of AI, has three categories: supervised learning, unsupervised learning, and reinforcement learning. Supervised learning needs labeled data for the training of the model while unsupervised learning and reinforcement learning are used for the unlabeled data. Deep learning is a subfield of machine learning which uses neural networks inspired by biological neurons. Deep learning extracts features from the data. Moreover, it needs a large amount of data compared to conventional machine learning. Artificial intelligence (AI) is the main technology that will help drive the development, help, and aid in the realization of the idea of the metaverse. AI algorithms (i.e., machine learning, deep learning, reinforcement learning, computer vision, etc.) are the “key” to connecting the virtual world and the real world [60]. By using artificial intelligence technologies, the metaverse can safely and freely participate in social and economic activities beyond the boundaries of the real world [61].

### 3.7. Digital Twin

The digital twin refers to a digital representation of physical objects [62]. The digital replica of the real objects is created in the virtual world. It has been used in many fields such as healthcare, manufacturing, and smart cities. This technology has three main components: the real object, its digital replica or virtual equivalent, and the connection of data between the physical and virtual object. In the healthcare sector, the digital twin played an important role during the COVID-19 pandemic. It enabled doctors to monitor patients virtually while tracking their vital signs such as blood pressure, heart rate, and temperature through wearable devices such as smartphones, etc. [63].

### 3.8. Computer Vision

Computer vision enables the computer to observe and see objects. It allows XR devices to identify and understand visual information of the real-world environment assisting to create virtual environments. This technology is vastly used in AR/VR and XR applications for the reconstruction of 3D objects in cyberspace. In the metaverse, computer vision-based algorithms are being used to build a better and more accurate user environment in a 3D immersive environment [64]. Computer vision can perform object detection, object classification, object segmentation, and object localization with high accuracy using its latest algorithms such as Mask RCNN, YOLO, etc. [65]. Action recognition and gesture recognition, which are also very important aspects in the metaverse, are also possible using computer vision because avatars must recognize and understand the actions of other avatars [66,67,68].

### 3.9. Blockchain

Blockchain technology is one of the indispensable technologies that make the metaverse ecosystem invincible. Blockchain was introduced by Nakamoto Satoshi for the first time in 2008 [69]. Blockchain is also known as a distributed ledger, and it consists of consecutive blocks linked with each other bearing the hash value of the previous block header [70]. It stores information in a decentralized network with digital signatures. The stored information is not subject to change. Blockchain is mainly used in the financial domain; however, it can be used in other areas such as risk management, healthcare, education, asset tracking, cyber security, and social services [71]. In healthcare, metaverse blockchain can be used for the confidentiality of patients’ data. Thus, enhancing the security of the patient’s record. Once the patient’s data are stored in the blockchain, they cannot be altered or discarded. It also helps manage electronic medical records and provides faster and cheaper patient care. Moreover, it provides interoperability, integrity, data security, and data exchange facilities [72]. Blockchain technology manages all the interactions between the physical and virtual environment with improved network security. Additionally, all the interactions are also recorded to keep track of them. This capability of blockchain helps doctors to go through and quickly understand the medical history of patients.

### 3.10. Avatar

The word “Avatar” is a Hindi concept that means the incarnation of the Hindu god in the ordinary world in the form of a human or animal. In the metaverse, humans appear as avatars in the virtual world which are the actual replica of human beings. Users can modify the look or the overall appearance of their avatars according to their own choice. A typical example is a scenario in advanced games such as Fortnite. In healthcare, avatars have been used to provide patient monitoring, treatment, diagnostic services, and educational training for doctors, nurses, and other healthcare staff [73,74]. It provides better visualization in a virtual environment thus helping surgeons to perform complex procedures seamlessly.

## 4. The Proposed Architecture of Healthcare Metaverse Powered by Explainable Artificial Intelligence (XAI) and Blockchain (BC)

**Figure 2:**
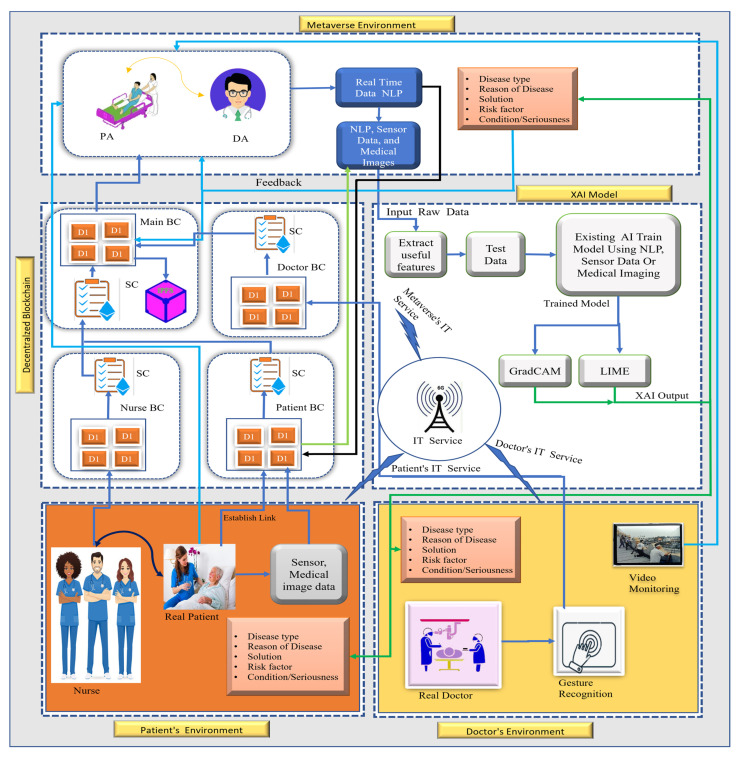
The architecture of the healthcare metaverse. **Note**: DA denotes doctor’s avatar, PA: patient avatar, CG: caregiver avatar, BC: blockchain, SC: smart contract, VH: virtual hospital, and OT: operating theatre.

In this section, we present our proposed framework for the healthcare metaverse. This architecture is powered by artificial intelligence and blockchain technology. Figure 2 shows the overall architecture of our proposed study. The architecture comprises three environments: (i) the doctor or physician environment, (ii) the metaverse or virtual environment, and (iii) the patient environment. The main idea behind this study is to provide virtual health services assisted by artificial intelligence and blockchain with immersive experiences to patients smoothly. Thanks to the latest technologies such as artificial intelligence, blockchain, and metaverse, which make it possible to diagnose and perform complex medical procedures by providing tremendous medical services virtually. Patients can receive counseling and other healthcare facilities through metaverse technology. Artificial intelligence technology is incorporated for diagnosing diseases, predicting, and monitoring patients’ health conditions. The artificial-intelligence-based model also assists doctors to perform complex procedures with minimal error. In the specific case of a medical surgical procedure, the AI-based model enables segmentation and annotations helping the surgeons to overlay their hands on the affected part thus improving the accuracy of the procedure. To ensure data security, patients’ data are acquired, stored, and transferred using blockchain technology because the data cannot be altered and misused by any person. Furthermore, it enables transparency and traceability while developing trust among the patients. The doctor and patient are assigned an ID to keep track of their identity, maintain medical history, and recognize them uniquely.

### 4.1. Doctor’s Environment

Doctors or physicians enter this environment remotely and become part of it. The doctor interacts with the virtual environment using XR tools and all the records are stored on the blockchain. The doctor needs to register first and then an ID is assigned to them which is stored on the blockchain and interaction with the virtual environment occurs via this ID. A block representing a transaction is created when the doctor enters the environment.

(1) Tn=[IDc, tnd, T, Sig(D)]

where *Tn* denotes transaction, *IDc* denotes the *ID* of the chain code, *tnd* denotes the transaction payload, T denotes the time of treatment, and the digital signature of the doctor is denoted by *Sig(D).*

### 4.2. Patient’s Environment

In the patient’s environment, eventual healthcare facilities will be available. These facilities include nurses, caregivers, and cobots for the patients’ treatment process. When a patient requests treatment, he needs to go through a registration process. The registration information will be stored on the blockchain. A caregiver is assigned to the patient after analyzing the disease condition. The patient will access the virtual environment with the patient’s ID using VR tools. Likewise, when a patient registers with an ID as *IDp* and requests treatment, then a block is created as:

(2) Tn=[IDc, tnp, T, Sig(P)]

where *Tn* denotes transaction, *IDc* denotes the *ID* of the chain code, *tnp* denotes the transaction payload, T denotes the time of treatment, and the digital signature of the patient is denoted by *Sig(P).*

Likewise, nurses or caregivers are also assigned to the patient when the patient is registered and assigned a doctor for treatment. The transaction block for the caregiver is also created as shown in Equation (3).

(3) Tn=[IDc, tncg, T, Sig(cg)]

where *Tn* denotes transaction, *IDc* denotes the *ID* of the chain code, *tncg* denotes the transaction payload of the caregiver, T denotes the time of treatment, and the digital signature of the caregiver is denoted by *Sig(cg).*

### 4.3. Metaverse Environment

The metaverse environment is the main part of the whole architecture as all the healthcare services will be provided in this environment virtually. Avatars of patients, medical staff, and doctors are created in this environment. The consultation process begins between the doctor and the patient through their respective avatars in the metaverse. During the consultation process, voices are recorded, and data are extracted using natural language processing (NLP). After analyzing the NLP data, if there is a need for more information related to the disease of the patient such as image data (CT scans or MRI) or other clinical data, the patient will provide the required laboratory test reports from the specialists. For example, in the case of a patient with a lung disease the physician may ask the patient for a CT or MRI of the chest from a radiologist and that data (CT scans or MRI) provided by the radiologist will be transferred to the patient’s blockchain thus accessible to the physician.

After data collection, we use a pre-trained AI model on which we shall test collected data for predictions. Explainable artificial intelligence (XAI) approaches, i.e., LIME/GradCAM, can be used depending on the type of data (image data) or the nature of the intervention (surgical procedure). XAI enables the doctors or physicians to determine those factors which account for the disease of the patients clearly. Moreover, the explainable artificial-intelligence-based model will ensure the trust, explainability, interpretability, and transparency of the model by providing logical reasoning for the prediction. The said output is available both in the doctor/patient’s environment and the metaverse. After the prediction of the disease, the patient is treated accordingly. In a specific case, such as that of a surgical procedure, a cobot is needed and instructions are sent to the patient’s environment for a surgical operation to be performed on the patient. A gesture recognition module is used to collect doctors’ real-time movement to the cobots, the operation is supported by video monitoring and real OT setup. Algorithm 1 shows the workflow of artificial intelligence technology in our proposed architecture.
**Algorithm 1:** Artificial-intelligence-based model for the healthcare metaverse*Input: NLP data, Sensor data, Image data, Trained model* *Output: O(XAI)**1.**Procedure AI_model(NLP data, Sensor data, Image data, Trained model)**2.**Data←load_data(NLP_data, sensor_data, Image_data)**3.**Data←Data_preprocessing(Data)**4.**Prediction←Trained_model.predict(Data)**5.**If (Data include medical images) then* *Import GradCAM* *Heatmap=make_gradcam_heatmap(Trained_model, Data)* *Else: Import LIME* *e = LIME.GradientExplainer(Trained_model, Data)* *LIME_values = e.LIME_values(Data)**6.**Display_gradcam/LIME**7.**O(XAI)←LIME.image_plot(LIME_values)* *O(XAI)←display_gradcam(heatmap)**8.**End.*

In Algorithm 1, data from different environments are collected in the form of images and CSV format. The artificial-intelligence-based model evaluates the data and makes a prediction using the GradCAM/LIME. This technique addresses the black-box nature of the AI models. It ensures the transparency and explainability of the model. The output of the AI model could be used by medical professionals for treatment decisions and complex procedures providing a better immersive experience.

Algorithm 2 shows the information flow on the blockchain in our metaverse. In Algorithm 2, when a patient requests a consultation with a doctor, the system searches for the most suitable and available doctor. If the patient is already registered on the blockchain in the metaverse healthcare hospital then patient information is retrieved; however, the non-registered patients will have to go through the registration process. A caregiver is assigned to the patient. Furthermore, the patient’s and caregiver’s smart contracts will be executed. If the doctor is available and his expertise meets the patient’s treatment request, he needs to approve the request of the patient by signing the request. With the signature of the doctor, the smart contract of the doctor will be executed. The treatment process will begin in the virtual environment identified by *ID_VEnv._* Once the treatment is completed the output of the XAI is sent onto the blockchains of respective environments and the environment is destroyed.
**Algorithm 2:** Blockchain-based network for the healthcare metaverse*Input: Patient P, Doctor D, Consultation query Cq, Caregiver Cg; and Prediction output O(XAI)* *Output: Initiate treatment in metaverse ID_VEnv_ (Virtual Environment) and activate patient environment*1.*Procedure: Blockchain_metahealth()*2.*For Cq by P do* *Response←Fetch_suitable(D)* *If (ID_P_∈BC_P_) then * *Assign Caregiver (BC_Cg_) * *Else * *Display (“Patient does not exist, please register”)* *End If* *End for*3.*Execute_Contract (ID_P_, tnp, Sig(P))* *Execute_Contract (ID_Cg_, tncg, Sig (Cg))*4.*If Dk←Sig (D) then * *Execute−Contract (ID_D_, tnd)* *Setup VEnv←(ID_P_, ID_D_, ID_Cg_, ID_VEnv_)* *Execute−Contract (ID_VEnv_)* *Main BC←O(XAI)* *Order Tn on Main BC (IDp, tnp, T, Sig(P)←Verify (Sig (D), Tn, D)* *Order Tn on Main BC (IDp, tnp, T, Sig(P)←Verify (Sig (cg), Tn, Cg)* *End If*5.*End.*
***Note:*** *ID_P_ denotes Id of patient, BC_P_: blockchain of patient, BC_Cg_: blockchain of caregiver, ID_Cg_: Id of caregiver, tncg: transaction pay load of caregiver, ID_D_: Id of doctor, ID_VEnv_: Id of virtual environment.*

Table 2 shows the comparison of some related works with our framework for the metaverse in healthcare. Our study is based on detailed information and usage of different technologies, i.e., XAI (Grad-CAM, LIME) and blockchain (decentralized BC), as compared to the previous studies. Furthermore, our framework supports a variety of data types for instance medical image data, NLP-based data, sensors, and other clinical data.

## 5. Metaverse Healthcare Advantages and Challenges

**Figure 3:**
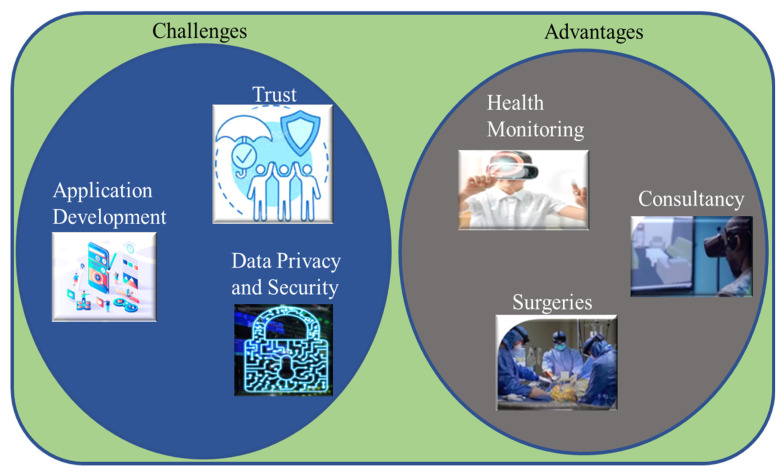
Challenges and advantages of metaverse in healthcare.

Although the metaverse has many advantages, there are several challenges as well. In this section, we elucidate some of those advantages and challenges. Figure 3 shows some of those challenges and advantages.

### 5.1. Advantages of Metaverse in Healthcare

#### 5.1.1. Health Monitoring

Patients can monitor their health using AI-based systems in the metaverse and it will help them to keep track of their health without taking any appointments with doctors or visiting any hospital physically.

#### 5.1.2. Consultation in an Immersive Environment

The patients could be able to consult with doctors or medical professionals virtually in a surreal environment using virtual reality technology.

#### 5.1.3. Surgeries in a Virtual Environment

The advancement in metaverse technology enables doctors and surgeons to perform surgeries virtually with high accuracy and minimal human error. Surgical training can also be conducted using metaverse. Seoul National University Bundang hospital offered training on lung cancer surgery through a metaverse. This training was received by more than 200 surgeons [23].

### 5.2. Challenges of Metaverse in Healthcare

#### 5.2.1. Application Development

Since metaverse is a multidisciplinary technology that involves so many other technologies such as cloud computing, edge computing, networking, AR, VR, XR, AI, avatars, blockchain, 3D rendering of each object, etc. Therefore it is a challenging task to develop a metaverse world that can provide a surreal experience. It needs high proficiency, resources, and skills. The integration and coordination of all the technologies should be seamless and efficient.

#### 5.2.2. Data Privacy and Security

The metaverse is highly vulnerable to data privacy and security because the whole physical world is digitized to create a replica in the metaverse environment. Data security is a very important factor in the metaverse ecosystem. Therefore, protecting the metaverse ecosystem from potential threats is a big challenge. User privacy, such as geographical privacy, habit, living styles, etc., may be violated while engaging in digital lives in the metaverse during the life-cycle of data services, which includes data perception, transmission, processing, governance, and storage. If the metaverse-enabling devices for example Oculus helmet and Meta Quest are attacked by hackers this may result in serious consequences [77]. Most healthcare IoT systems are based on centralized approaches and they pose several potential threats. For example, they present security and robustness flaws including a single point of failure (and single point of breach) and unintentional or malicious record modification. Moreover, there are several other security and privacy challenges as highlighted by YK Dwivedi et al. [78] in their study. To ensure the privacy and security of patients’ data blockchain technology has been introduced to make it invincible. Many experts endorse the vulnerability of IoT-based health systems and consider blockchain technology as a viable solution to potential threats [79]. Blockchain has several inherent features such as decentralized applications, immutability, trust, transparency, and provenance [80]. These characteristics of blockchain provide unbreachable security to healthcare data. Many researchers proposed different blockchain-based approaches to provide security to healthcare data and healthcare IoTs [81,82]. For example, Zeeshan Zulkifli et al. [83] presented a new architecture that is based on fuzzy and blockchain enabling privacy and quick response facilities to the healthcare IoTs. By leveraging fuzzy logic, they suggest a behavior-driven adaptive security mechanism for healthcare IoTs and blockchain-based networks. They also give a heuristic method to behavior-driven adaptive security that offers Authentication, Authorization, and Audit Logs (AAA) services. TT Kuo et al. [84] proposed a decentralized blockchain to uphold privacy for healthcare predictive models. They incorporated privacy online machine learning with blockchain. This approach helped them to enhance the security and robustness of the healthcare system.

#### 5.2.3. Trusts

Since the metaverse is emerging and is a nascent field, people do not have much confidence in the healthcare metaverse. People do believe in doctors rather than rely on avatars and hologram AI-based systems. The trust deficit between the patients and the metaverse should be reduced by improving the quality of metaverse healthcare services, treatments, and diagnostic capabilities [85]. Blockchain and explainable AI technologies help improve trust among users. As far as data security and transparency is concerned, blockchain provides the best solutions because of its immutable and distributed nature. In the case of healthcare, the patients have more trust in such systems and they are the owner of their data. Their medical records could not be breached or tampered with easily. Furthermore, it also minimizes treatment negligence from doctors because they will be held accountable for their prescriptions since the treatment history on the blockchain is immutable and can be traced easily. Ultimately it provides transparency and enhances trust [86]. Besides blockchain technology, a patient-centric interaction policy also helps improve trust among patients [87,88].

## 6. Conclusions

The advancement in the metaverse will open new avenues in the healthcare sector bringing innovation and improvement in this domain. The metaverse ecosystem stands on the base technologies such as artificial intelligence, blockchain, 5G, virtual reality, and digital twin and these are the building blocks of the metaverse. In this research work, we proposed a metaverse ecosystem for healthcare incorporating artificial intelligence and blockchain. It consists of three environments, namely, the doctor’s environment, the metaverse environment, and the patient’s environment. The patient and doctor enter the metaverse environment from their respective domains using AR, VR, and XR technologies. A metaverse healthcare environment provides an immersive experience for doctors and patients in a virtual world. Digital avatars are used by doctors and patients to interact with each other and to generate and exchange information. In the virtual hospital/ metaverse environment the patients would receive several kinds of health facilities such as treatment, surgery, counseling, consultancy, therapy, etc. The data of the patients will be sent to the explainable artificial intelligence-based models (XAI) for analysis, prediction, and diagnosis of diseases. The XAI models will provide the logical reasoning for the disease and its prediction. The medical experts examine the prediction and provide treatments to the patients accordingly. In case of surgery, the surgeon will perform the surgery in the metaverse, and it will be reflected in the patient environment on the patient through cobots and other necessary technologies; blockchain technology ensures the security and privacy of patients’ data which also helps to keep track of the medical history of the patients. The use of blockchain and artificial intelligence enables the metaverse to provide a safe environment for the users to avail of its services. This proposed metaverse-based healthcare system could be used for multiple purposes such as training and education of healthcare professionals, diagnostics, therapeutics, and other healthcare services leveraging the healthcare sector for the betterment of mankind.

## Figures and Tables

**Table 1:** Comparative analysis of some of the technologies and use cases.

	Description	Technology	Use Case	Reference
1	Avatar face recognition	Machine learning model with Markov random field.	Face recognition of the avatar.	[33]
2	Metaverse and virtual healthcare	The mixture of AI, IoT, blockchain, and AR/VR/MR	Metaverse in ophthalmology.	[12]
3	Smart healthcare in the metaverse	AI, blockchain, and wearable technology.	Chronic disease treatment.	[34]
4	Data collection in metaverse from IoT devices	Digital twins, IoT, and networking.	Virtual services with real-life IoT data.	[35]
5	Lung cancer surgery in the metaverse	Extended reality, virtual reality, and augmented reality.	Lung cancer surgery assisted by VR.	[23]
6	Adoption of the metaverse in spine care. A view of education, diagnosis, consultation, surgery, and research perspectives	Artificial intelligence, virtual reality, and robotics.	Healthcare, education, and research.	[36]
7	Diagnosis and prevention of cardiovascular diseases	Virtual reality and augmented reality.	Metaverse-assisted cardiovascular diseases treatment.	[21]
8	Healthcare education, clinical treatment, physical fitness, and mental wellness in the metaverse	Virtual reality and augmented reality, mixed reality, and digital twins.	Telemedicine, healthcare education in the metaverse.	[37]
9	Healthcare simulation in the virtual world	Virtual reality.	Simulation in the virtual world.	[38]

**Table 2:** Comparison of our architecture with other studies in the healthcare metaverse.

Ref.	AI		Blockchain		Input Data Types		
	XAI/Grad-CAM	XAI/LIME	Transaction Info.	Decentralized Technology	Medical Image Based	NLP Based	Sensor/Clinical Based
[75]	×	×	√	√	×	√	√
[76]	×	×	×	×	√	×	×
**Ours**	**√**	**√**	**√**	**√**	**√**	**√**	**√**

Note: √ denotes Yes; × denotes No.
